# DIF Statistical Inference Without Knowing Anchoring Items

**DOI:** 10.1007/s11336-023-09930-9

**Published:** 2023-08-07

**Authors:** Yunxiao Chen, Chengcheng Li, Jing Ouyang, Gongjun Xu

**Affiliations:** 1 London School of Economics and Political Science, London, UK; 2 University of Michigan, Ann Arbor, USA

**Keywords:** differential item functioning, measurement invariance, item response theory, least absolute deviations, confidence interval

## Abstract

Establishing the invariance property of an instrument (e.g., a questionnaire or test) is a key step for establishing its measurement validity. Measurement invariance is typically assessed by differential item functioning (DIF) analysis, i.e., detecting DIF items whose response distribution depends not only on the latent trait measured by the instrument but also on the group membership. DIF analysis is confounded by the group difference in the latent trait distributions. Many DIF analyses require knowing several anchor items that are DIF-free in order to draw inferences on whether each of the rest is a DIF item, where the anchor items are used to identify the latent trait distributions. When no prior information on anchor items is available, or some anchor items are misspecified, item purification methods and regularized estimation methods can be used. The former iteratively purifies the anchor set by a stepwise model selection procedure, and the latter selects the DIF-free items by a LASSO-type regularization approach. Unfortunately, unlike the methods based on a correctly specified anchor set, these methods are not guaranteed to provide valid statistical inference (e.g., confidence intervals and *p*-values). In this paper, we propose a new method for DIF analysis under a multiple indicators and multiple causes (MIMIC) model for DIF. This method adopts a minimal $L_1$ norm condition for identifying the latent trait distributions. Without requiring prior knowledge about an anchor set, it can accurately estimate the DIF effects of individual items and further draw valid statistical inferences for quantifying the uncertainty. Specifically, the inference results allow us to control the type-I error for DIF detection, which may not be possible with item purification and regularized estimation methods. We conduct simulation studies to evaluate the performance of the proposed method and compare it with the anchor-set-based likelihood ratio test approach and the LASSO approach. The proposed method is applied to analysing the three personality scales of the Eysenck personality questionnaire-revised (EPQ-R).

**Supplementary Information:**

The online version contains supplementary material available at 10.1007/s11336-023-09930-9.

## Introduction

Measurement invariance refers to the psychometric equivalence of an instrument (e.g., a questionnaire or test) across several specified groups, such as gender and ethnicity. The lack of measurement invariance suggests that the instrument has different structures or meanings to different groups, leading to biases in measurements (Millsap, 2012).

Measurement invariance is typically assessed by differential item functioning (DIF) analysis of item response data that aims to detect the measurement non-invariant items (i.e. DIF items). More precisely, a DIF item has a response distribution that depends on not only the latent trait measured by the instrument but also respondents’ group membership. Therefore, the detection of a DIF item involves comparing the item responses of different groups, conditioning on the latent traits. The complexity of the problem lies in that individuals’ latent trait levels cannot be directly observed but are measured by the instrument that may contain DIF items. In addition, different groups may have different latent trait distributions. This problem thus involves identifying the latent trait and then conducting the group comparison given individuals’ latent trait levels.

Many statistical methods have been developed for DIF analysis. Traditional methods for DIF analysis require prior knowledge about a set of DIF-free items, which is known as the anchor set. This anchor set is used to identify the latent trait distribution. These methods can be classified into two categories. Methods in the first category (Mantel and Haenszel, 1959; Dorans and Kulick, 1986; Swaminathan and Rogers, 1990; Shealy and Stout, 1993; Zwick et al., 2000; Zwick and Thayer, 2002; May, 2006; Soares et al., 2009; Frick et al., 2015) do not explicitly assume an item response theory (IRT) model, and methods in the second category (Thissen, 1988; Lord, 1980; Kim et al., 1995; Raju, 1988, 1990; Woods et al., 2013; Oort, 1998; Steenkamp and Baumgartner, 1998; Cao et al., 2017; Woods et al., 2013; Tay et al., 2015, 2016) are developed based on IRT models. Compared with non-IRT-based methods, an IRT-based method defines the DIF problem more clearly, at the price of potential model misspecification. Specifically, an IRT model represents the latent trait as a latent variable and further characterizes the item-specific DIF effects by modelling each item response distribution as a function of the latent variable and group membership.

The DIF problem is well-characterized by a multiple indicators, multiple causes (MIMIC) IRT model, which is a structural equation model originally developed for continuous indicators (Zellner, 1970; Goldberger, 1972) and later extended to categorical item response data (Muthen, 1985; Muthen et al., 1991; Muthen and Lehman, 1985). A MIMIC model for DIF consists of a measurement component and a structural component. The measurement component models how the item responses depend on the measured psychological trait and respondents’ group membership. The structural component models the group-specific distributions of the psychological trait. The anchor set imposes zero constraints on item-specific parameters in the measurement component, making the model, including the latent trait distribution, identifiable. Consequently, the DIF effects of the rest of the items can be tested by drawing statistical inferences on the corresponding parameters under the identified model.

Anchor-set-based methods rely heavily on a correctly specified set of DIF-free items. The misspecification of some anchor items can lead to invalid statistical inference results – Type I errors increase and power decreases when anchor items are not completely DIF-free (Kopf et al., 2015b). To address this issue, item purification methods (Candell and Drasgow, 1988; Clauser et al., 1993; Fidalgo et al., 2000; Wang and Yeh, 2003; Wang and Su, 2004; Wang et al., 2009; Kopf et al., 2015b, a) have been proposed that iteratively select an anchor set by stepwise model selection methods. Several recently developed tree-based DIF detection methods (Strobl et al., 2015; Tutz and Berger, 2016; Bollmann et al., 2018), which can detect DIF brought by continuous covariates, may be viewed as item purification methods. However, with multiple items containing DIF, item purification may suffer from masking and swamping effects (Barnett and Lewis, 1994). More recently, regularized estimation methods (Magis et al., 2015; Tutz and Schauberger, 2015; Huang, 2018; Belzak and Bauer, 2020; Bauer et al., 2020; Schauberger and Mair, 2020) have been proposed that use LASSO-type regularized estimation procedures for simultaneous model selection and parameter estimation. Moreover, Bechger and Maris (2015) proposed DIF detection methods based on the idea of differential item pair functioning, which does not require prior information about anchor items. Based on a similar idea as in Bechger and Maris (2015), Yuan et al. (2021) proposed a relative change of difficulty difference method, in which data visualisation tools and Monte Carlo simulations are used to detect DIF items. Unfortunately, unlike many anchor-set-based methods with a correctly specified anchor set, these methods do not provide valid statistical inference for separately testing the null hypothesis of “item *j* is DIF-free” for each individual item *j*. Consequently, the type-I error for testing the hypothesis cannot be guaranteed to be controlled at a pre-specified significance level. For example, some item purification methods proceed by performing one or multiple hypothesis tests in each iteration, yielding some item-specific *P*-values. However, these tests are performed conditioning on the model previously selected, which fails to adjust for uncertainty in the iterative selection process (noting that the same data are used repeatedly). Consequently, the obtained *P*-values are not guaranteed to follow a uniform distribution, even in an asymptotic sense. Yuan et al. (2021) constructed confidence intervals for individual items. However, these confidence intervals are constructed by simulating from a setting where all the items are DIF-free. Consequently, they may not have the desired coverage or, equivalently, yield valid *P*-values for each individual item when there exist DIF items. Furthermore, although the regularised estimation methods have been shown to accurately detect DIF items, they are typically computationally intensive since they involve solving multiple regularized maximum likelihood estimation problems with different tuning parameters.

This paper proposes a new method that addresses the aforementioned issues with the existing methods. The proposed method can statistically accurately and computationally efficiently estimate the DIF effects without requiring prior knowledge about anchor items. It draws statistical inferences on the DIF effects of individual items, yielding valid confidence intervals and *P*-values. The point estimation and statistical inference lead to accurate detection of the DIF items, for which the item-level type-I error and further some test-level risk (e.g., false discovery rate) can be controlled by the inference results. The method is proposed under a MIMIC model with a two-parameter logistic (Birnbaum, 1968) IRT measurement model and a linear structural model. The key to this method is a minimal $L_1$ norm condition for identifying the true model. This condition assumes that the DIF effect parameters of the true model are sparse and, thus, imposes a sensible global structure on the measurement model. This structure can effectively identify the latent trait without knowing the anchor items and further detect the DIF items. As will be shown later, the minimal $L_1$ norm condition holds when the proportion of non-DIF items is sufficiently large. Methods are developed for estimating the model parameters and obtaining confidence intervals and *p*-values, where the method for obtaining the confidence intervals and *p*-values can be viewed as a parametric bootstrap procedure (Davison and Hinkley, 1997; Zhang, 2018). Procedures for the detection of DIF items are further developed. Our method is compared to the likelihood ratio test method (Thissen et al., 1993) that requires an anchor set, and a recently proposed LASSO-based approach (Belzak and Bauer, 2020).

The rest of the paper is organised as follows: In Sect. 2, we introduce a MIMIC model framework for DIF analysis. Under this model framework, a new method is proposed for the statistical inference of DIF effects in Sect. 3. Related works are discussed in Sect. 4. Simulation studies and a real data application are given in Sects. 5 and 6, respectively. We conclude with discussions in Sect. 7. All the proofs for the proposition and theorems presented in the article, and the implementation details of the proposed algorithms can be found in the Supplementary Materials.

## A MIMIC Formulation of DIF

Consider *N* individuals answering *J* items. Let $Y_{ij} \in \{0, 1\}$ be a binary random variable, denoting individual *i*’s response to item *j*. Let $y_{ij}$ be the observed value, i.e., the realization, of $Y_{ij}$. For the ease of exposition, we use ${\textbf{Y}}_i = (Y_{i1}, \ldots , Y_{iJ})$ to denote the response vector of individual *i*. The individuals are from two groups, indicated by $x_i = 0, 1$, where 0 and 1 are referred to as the reference and focal groups, respectively. We further introduce a latent variable $\theta _i$, which represents the latent trait that the items are designed to measure. DIF occurs when the distribution of $\textbf{Y}_i$ depends on not only $\theta _i$ but also $x_i$. More precisely, DIF occurs if ${\textbf{Y}}_i$ is not conditionally independent of $x_i$, given $\theta _i$. Seemingly a simple group comparison problem, DIF analysis is non-trivial due to the latency of $\theta _i$. In particular, the distribution of $\theta _i$ may depend on the value of $x_i$, which confounds the DIF analysis. In what follows, we describe a MIMIC model framework for DIF analysis, under which the relationship among ${\textbf{Y}}_i$, $\theta _i$, and $x_i$ is characterized. It is worth pointing out that this framework can be generalized to account for more complex DIF situations; see more details in Sect. 4.

### Measurement Model

The two-parameter logistic (2PL) model (Birnbaum, 1968) is widely used to model binary item responses (e.g., wrong/right or absent/present). In the absence of DIF, the 2PL model assumes a logistic relationship between $Y_{ij}$ and $\theta _i$, which is independent of the value of $x_i$. That is,

1 $$\begin{aligned} P(Y_{ij} = 1\vert \theta _i = \theta ) = \frac{\exp (a_j\theta + d_j)}{1+\exp (a_j\theta + d_j)}, \end{aligned}$$

where the slope parameter $a_j$ and intercept parameter $d_j$ are typically known as the discrimination and easiness parameters, respectively. The right-hand side of (1) as a function of $\theta$ is known as the 2PL item response function. When the items potentially suffer from DIF, the item response functions may depend on the group membership $x_i$. In that case, the item response function can be modelled as:

2 $$\begin{aligned} P(Y_{ij} = 1\vert \theta _i = \theta , x_i) = \frac{\exp (a_j\theta + d_j + \gamma _j x_i)}{1+\exp (a_j\theta + d_j + \gamma _j x_i)}, \end{aligned}$$

where $\gamma _j$ is an item-specific parameter that characterizes its DIF effect. More precisely,

$$\begin{aligned} \frac{P(Y_{ij} = 1\vert \theta _i = \theta , x_i = 1)/(1- P(Y_{ij} = 1\vert \theta _i = \theta , x_i = 1))}{P(Y_{ij} = 1\vert \theta _i = \theta , x_i=0)/(1-P(Y_{ij} = 1\vert \theta _i = \theta , x_i=0))} = \exp (\gamma _j). \end{aligned}$$

That is, $\exp (\gamma _j)$ is the odds ratio for comparing two individuals from two groups who have the same latent trait level. Item *j* is DIF-free under this model when $\gamma _j = 0$. We further make the local independence assumption as in most IRT models. That is, $Y_{i1}$, ..., $Y_{iJ}$ are assumed to be conditionally independent, given $\theta _i$ and $x_i$.

### Structural Model

A structural model specifies the distribution of $\theta _i$, which may depend on the group membership. Specifically, we assume the conditional distribution of $\theta _i$ given $x_i$ to follow a normal distribution,

$$\begin{aligned} \theta _i \vert x_i \sim N(\beta x_i, 1_{\{x_i = 0\}} + \sigma ^2 1_{\{x_i = 1\}}). \end{aligned}$$

Note that the latent trait distribution for the reference group is set to a standard normal distribution to identify the location and scale of the latent trait. A similar assumption is typically adopted in IRT models for a single group of individuals.

**Fig. 1:**
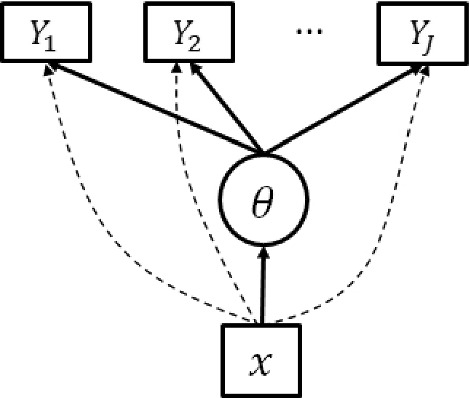
The path diagram of a MIMIC model for DIF analysis. The subscript *i* is omitted for simplicity. The dashed lines from *x* to $Y_j$ indicate the DIF effects.

The MIMIC model for DIF combines the above measurement and structural models, for which a path diagram is given in Fig. 1. The marginal likelihood function for this MIMIC model takes the form

3 $$\begin{aligned} L(\Xi ) = \prod _{i=1}^N \int \left( \prod _{j=1}^J \frac{\exp (y_{ij}(a_j\theta + d_j + \gamma _j x_i))}{1+\exp (a_j\theta + d_j + \gamma _j x_i)}\right) \frac{1}{\sqrt{2\pi }} \exp \left( \frac{-(\theta - \beta x_i)^2}{2(1_{\{x_i = 0\}} + \sigma ^2 1_{\{x_i = 1\}})}\right) d\theta ,\qquad \end{aligned}$$

where $\Xi = \{\beta , \sigma ^2, a_j, d_j, \gamma _j, j = 1, \ldots , J\}$ denotes all the fixed model parameters.

The goal of DIF analysis is to detect the DIF items, i.e., the items for which $\gamma _j \ne 0$. Unfortunately, without further assumptions, this problem is ill-posed due to the non-identifiability of the model. We discuss this identifiability issue below.

### Model Identifiability

Without further assumptions, the above MIMIC model is not identifiable. That is, for any constant *c*, the model remains equivalent, if we simultaneously replace $\beta$ and $\gamma _j$ by $\beta +c$ and $\gamma _j -a_jc$, respectively, and keep $a_j$ unchanged. This identifiability issue is due to that all the items are allowed to suffer from DIF, resulting in an unidentified latent trait. In other words, without further assumptions, it is impossible to disentangle the DIF effects and the difference between the latent trait distributions of the two groups.

According to Theorem 8.3 of San Martín (2016), the location shift described above is the only source of indeterminacy for this MIMIC model when $J\ge 3$ and the sizes of both groups go to infinity. Let $\Xi ^* = \{\beta ^*, (\sigma ^*)^2, a_j^*, d_j^*, \gamma _j^*, j = 1, \ldots , J\}$ be a set of parameters for the true model. Then for any constant *c*, the set of parameters $\Xi ^*(c) = \{\beta ^*+c, (\sigma ^*)^2, a_j^*, d_j^*, \gamma _j^* - a_j^*c, j = 1, \ldots , J\}$ gives the same data distribution. Moreover, if a set of parameters implies the same data distribution, then it has to take the form of $\Xi ^*(c)$ for some constant *c*. Knowing one or more anchor items means that the corresponding $\gamma _j^*$s are known to be zero, which fixes the location indeterminacy. However, if no anchor item is known, we need to answer the question: which member of this equivalent class should be used to define DIF effects? We address it in Sect. 3.

## Proposed Method

In what follows, we address the model identifiability problem raised above and then propose a new method for DIF analysis that does not require prior knowledge about anchor items under our definition of the true model parameters and additional regularity conditions. As will be shown in the rest, the proposed method can not only accurately detect the DIF items but also provide valid statistical inference for testing the hypotheses of $\gamma _j = 0$, for any $j = 1, \ldots , J$.

### Model Identifiability, Sparsity, and Minimal $L_1$ Condition

We now address the model identifiability problem. The most natural idea is to choose $\Xi ^*$ as the true parameter vector when the corresponding $\varvec{\gamma }^* = (\gamma _1^*, \ldots , \gamma _J^*)^\top$ is the sparsest in the equivalent class $\{\Xi ^*(c): c \in {\mathbb {R}}\}$. In other words, we say $\Xi ^*$ is the true model parameter when

4 $$\begin{aligned} \Vert \varvec{\gamma }^*\Vert _0 < \Vert \varvec{\gamma }^*(c)\Vert _0 \end{aligned}$$

for any $c\ne 0$, where $\varvec{\gamma }^*(c) = (\gamma _1^* - a_1^*c, \ldots , \gamma _J^* - a_J^*c)^\top$ and $\Vert \cdot \Vert _0$ denotes the $L_0$ norm, i.e., the number of nonzero entries in a vector. This definition requires the $\varvec{\gamma }^*$ to be unique, which further implies that $\Xi ^*$ is unique. We note that this sparsity assumption is essential if one wants to formulate the DIF detection problem as a model selection problem, i.e., using statistical criteria to decide which DIF effect parameters are zero. It is explicitly or implicitly made by most DIF detection methods that do not require anchor items, including item purification and regularised estimation methods. Note that our notion of true model parameter requires the true DIF parameter vector $\varvec{\gamma }^*$ to have at least two zero elements, i.e., $\Vert \varvec{\gamma }^*\Vert _0 \le J-2$. If $\Vert \varvec{\gamma }^*\Vert _0 \ge J-1$, then one can easily find a value of $c\ne 0$ such that $\Vert \varvec{\gamma }^*(c)\Vert _0 \le \Vert \varvec{\gamma }^*\Vert _0$ by setting $c = \gamma _j^*/a_j^*$ for any *j* satisfying $\gamma _j^* \ne 0$. In that case, the definition of $\Xi ^*$ is violated.

We also notice that the uniqueness of $\varvec{\gamma }^*$ is guaranteed when it is sufficiently sparse. In particular, when $\Vert \varvec{\gamma }^*\Vert _0 < J/2$, then $\Vert \varvec{\gamma }^*(c)\Vert _0 \ge J/2$ for any $c\ne 0$ (assuming that $a_j^* \ne 0$ for all *j*), and thus, the uniqueness of $\varvec{\gamma }^*$ is guaranteed. In the rest, we consider settings when $\varvec{\gamma }^*$ is sufficiently sparse. Further discussions will be provided in the sequel regarding the sparsity level. We note that this “sufficiently sparse" assumption aligns well with the practical utility of DIF analysis in educational testing (e.g., Holland & Wainer, 1993) as well as certain settings of psychological measurement (e.g., Chapter 1, Millsap, 2012) and health-related measurement (e.g., Scott et al., 2010). For example, in educational testing, DIF analysis is conducted to ensure the fairness of a test form. In this application, the test operator aims to identify a small number of DIF items that cause a bias in the test result. The identified items will be reviewed by domain experts and then revised or removed from the item pool. For this process to be operationally feasible, one typically needs to assume that most items are DIF-free, i.e., $\varvec{\gamma }^*$ is sufficiently sparse.

The $L_0$ norm is not easy to work with from a statistical perspective. Due to the randomness in the data, likelihood-based estimation methods almost never give us a truly sparse solution. Consequently, one essentially needs to search over $O(2^J)$ all possible models to find the sparest model (e.g., using a suitable information criterion). Item purification and regularized estimation methods narrow the search by stepwise procedures and regularized estimation procedures, respectively. Even with these methods, the computation can still be intensive, and consistent selection of the true model is not always guaranteed.

**Fig. 2:**
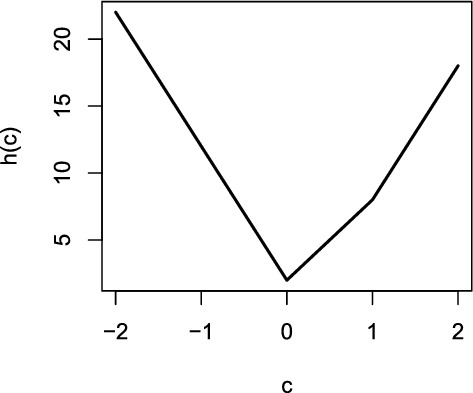
Function $h(c) = \sum _{j=1}^J \vert \gamma _j^* -a_j^*c\vert$, where $J = 10$, $a_j^* = 1$ for all *j*, $\gamma _j^* = 0$ and 1 for $j = 1, \ldots , 8$ and $j = 9, 10$, respectively. The minimal value of *h*(*c*) is achieved when $c = 0$.

To develop our method, we consider a surrogate to (4). Specifically, we require the following minimal $L_1$ (ML1) condition to hold

5 $$\begin{aligned} \sum _{j=1}^J \vert \gamma _j^* \vert < \sum _{j=1}^J \vert \gamma _j^* -a_j^*c\vert , \end{aligned}$$

or equivalently, $\Vert \varvec{\gamma }^*\Vert _1 < \Vert \varvec{\gamma }^*(c)\Vert _1$ for all $c \ne 0$. This assumption implies that, among all models that are equivalent to the true model, the true parameter vector $\varvec{\gamma }^*$ has the smallest $L_1$ norm. Equivalently, we can rewrite (5) as

6 $$\begin{aligned} \arg \min _c h(c) = 0, \end{aligned}$$

where $h(c) = \sum _{j=1}^J \vert \gamma _j^* -a_j^*c\vert$. We give an example of *h*(*c*) in Fig. 2, where *h*(*c*) is constructed with a sparse $\varvec{\gamma }^*$. More specifically, we construct *h*(*c*) with $J = 10$, $a_j^* = 1$ for all *j*, $\gamma _j^* = 0$ and 1 when $j = 1, \ldots , 8$ and $j = 9, 10$, respectively. In this example, we note that *h*(*c*) has a unique minumum at $c=0$, i.e., (5), or equivalently, (6) holds.

Although (4) and (5) are different in general, they coincide with each other when $\varvec{\gamma }^*$ is sufficiently sparse. The following proposition provides a sufficient and necessary condition for the ML1 condition (5) (or equivalently (6)) to hold. The proof is given in the Supplementary Materials.

#### Proposition 1

Assume that $a_j^* \ne 0$ for all *j*. Condition (5) holds if and only if

7 $$\begin{aligned} \sum _{j=1}^J |a_j^*| \left( - I\bigg (\frac{\gamma _j^*}{a_j^*} \ge 0\bigg ) + I\bigg (\frac{\gamma _j^*}{a_j^*}< 0\bigg )\right) < 0 \end{aligned}$$

and

8 $$\begin{aligned} \sum _{j=1}^J |a_j^*| \left( - I\bigg (\frac{\gamma _j^*}{a_j^*}> 0\bigg ) + I\bigg (\frac{\gamma _j^*}{a_j^*} \le 0\bigg )\right) > 0, \end{aligned}$$

where $I(\cdot )$ is the indicator function.

We note that inequalities (7) and (8) hold for the example in Fig. 2, where $\sum _{j=1}^J I(\gamma _j^* \ge 0) = 10$, $\sum _{j=1}^J I(\gamma _j^* < 0) =0$, $\sum _{j=1}^J I(\gamma _j^* \le 0) = 8$ and $\sum _{j=1}^J I(\gamma _j^* > 0) = 2$. To elaborate on the results of Proposition 1, we first consider a special case when $a_j^* = 1$ for all *j*, i.e., the measurement model is a one-parameter logistic model when there is no DIF. Then according to Proposition 1, the ML1 condition holds if and only if $\sum _{j=1}^J I(\gamma _j^* \ge 0) > \sum _{j=1}^J I(\gamma _j^* < 0)$ and $\sum _{j=1}^J I(\gamma _j^* \le 0)> \sum _{j=1}^J I(\gamma _j^* > 0).$ Suppose that more than half of the items are DIF-free, i.e., $\sum _{j=1}^J I(\gamma _j^* = 0) > J/2$. Then, the ML1 condition holds, because $\sum _{j=1}^J I(\gamma _j^* \ge 0)> J/2 > \sum _{j=1}^J I(\gamma _j^* < 0)$ and $\sum _{j=1}^J I(\gamma _j^* \le 0)> J/2> \sum _{j=1}^J I(\gamma _j^* > 0).$ In this case, as discussed previously, (4) also holds. More generally, let $\gamma _{(1)}^* \le \gamma _{(2)}^* \le \cdots \le \gamma _{(J)}^*$ be the order statistics of $\gamma _1^*$, ..., $\gamma _J^*$. The ML1 condition holds when $\gamma _{((J+1)/2)}^* = 0$ if *J* is an odd number, and when $\gamma _{(J/2)}^* = \gamma _{((J/2)+1)}^* = 0$ if *J* is an even number. That is, the ML1 condition holds when we have similar numbers of positive and negative DIF items and a few non-DIF items, in which case the ML1 condition can hold even if $\sum _{j=1}^J I(\gamma _j^* = 0) \le J/2$. However, if all the DIF items are of the same direction (all positive or all negative), then it is easy to show that the ML1 condition does not hold if $\sum _{j=1}^J I(\gamma _j^* = 0) \le J/2$.

We then extend the above discussion to the general setting where the discrimination parameters vary across items. Based on Proposition 1, we provide a sufficient condition for the ML1 condition, which suggests that the ML1 condition holds when $\gamma _j^* = 0$ for a sufficient number of items.

#### Corollary 1

Assume that $a_j^* \ne 0$ for all *j*. Let $\rho ^* = \max _j\{\vert a_j^*\vert \}/\min _j\{\vert a_j^*\vert \}$. Then Condition (5) holds if

9 $$\begin{aligned} \sum _{j=1}^J I(\gamma _j^*/a_j^* \le 0)> \rho ^* \sum _{j=1}^J I(\gamma _j^*/a_j^* > 0) \end{aligned}$$

and

10 $$\begin{aligned} \sum _{j=1}^J I(\gamma _j^*/a_j^* < 0) > \rho ^* \sum _{j=1}^J I(\gamma _j^*/a_j^* \ge 0). \end{aligned}$$

We note (9) and (10) are not a necessary condition, meaning that the ML1 condition can still hold even if (9) and (10) are not satisfied. Here, $\rho ^*$ quantifies the variation of the absolute discrimination parameters, where a larger value of $\rho ^*$ indicates a higher variation. Corollary 1 suggests that ML1 condition holds if $\sum _{j=1}^J I(\gamma _j^* = 0) > (\rho ^*/(1+\rho ^*))J$, in which case (4) also holds. For instance, when $\rho ^* = 2$, then the ML1 condition is guaranteed if $\sum _{j=1}^J I(\gamma _j^* = 0) > (2/3)J$, i.e., at least two-thirds of the items are DIF-free. This sparsity requirement can be relaxed if the sizes of items with $\gamma _j^*/a_j^* > 0$ and those with $\gamma _j^*/a_j^* < 0$ are balanced.

### Parameter Estimation

Suppose that the true model parameters satisfy the ML1 condition. Then, these parameters can be estimated by finding the ML1 estimate ${\hat{\Xi }} = \{{\hat{\beta }}, {\hat{\sigma }}^2, {\hat{\gamma }}_j, {\hat{d}}_j, {\hat{a}}_j, j=1, \ldots ,J\}$ satisfying

11 $$\begin{aligned} \log L({\hat{\Xi }}) = \max _{\Xi } \log L(\Xi ) \end{aligned}$$

and for any $\tilde{\Xi }= \{\tilde{\beta }, \tilde{\sigma }^2, \tilde{\gamma }_j, {\tilde{d}}_j, {\tilde{a}}_j, j=1, \ldots ,J\}$ satisfying $\log L(\tilde{\Xi }) = \max _{\Xi } \log L(\Xi )$,

12 $$\begin{aligned} \sum _{j=1}^J \vert {\hat{\gamma }}_j \vert \le \sum _{j=1}^J \vert \tilde{\gamma }_j\vert . \end{aligned}$$

That is, ${\hat{\Xi }}$ is a maximum likelihood estimate whose DIF parameter vector has the smallest $L_1$ norm. We adopt a two-stage estimator to find ${\hat{\Xi }}$. First, we find an estimator $\tilde{\Xi }$ that maximizes $\log L(\Xi )$, but the corresponding $\tilde{\varvec{\gamma }}$ not necessarily has the minimum $L_1$ norm in its equivalent class defined by location shift. Second, we find ${\hat{\Xi }}$ within the equivalent class of $\tilde{\Xi }$ such that the corresponding $\hat{\varvec{\gamma }}$ has the minimum $L_1$ norm.

In principle, $\tilde{\Xi }$ in the first stage can be obtained by maximizing $\log L(\Xi )$ without imposing constraints on model parameters. However, due to the location indeterminacy, the Hessian matrix of $\log L(\Xi )$ degenerates, and thus, the optimization often suffers from slow convergence. To avoid this issue, we fix the location indeterminacy issue by constraining $\tilde{\gamma }_1 = 0$. Due to the location indeterminacy of the model, one can always make this constraint without sacrificing the likelihood function value, even if item 1 is not DIF-free. We also remark that the constraint $\tilde{\gamma }_1 = 0$ can be replaced by any equivalent constraint, for example, $\tilde{\gamma }_2 = 0$, while not affecting the final estimation result. The two-stage estimator is summarized in Algorithm 1.

**Algorithm 1:**
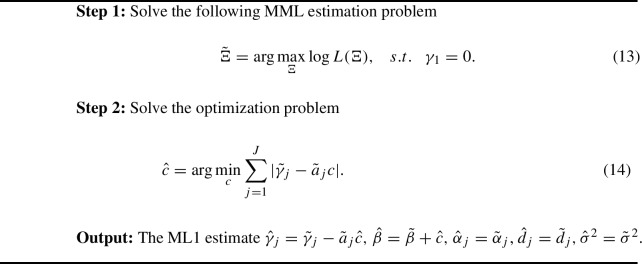
.

We provide some remarks about the optimisation in Step 2. This step finds the transformation that leads to the ML1 solution among all the models equivalent to the estimated model from Step 1. The optimization problem (14) is convex that takes the same form as the Least Absolute Deviations (LAD) objective function in median regression (Koenker, 2005). Specifically, the LAD function is a statistical optimization function measuring the sum of absolute residuals. Given a set of data $(x_i, y_i)$ for $i = 1,\dots , n$, the LAD function is defined as $S(f) = \sum _{i=1}^{n} \vert y_i - f(x_i) \vert$, and we seek to find *f* that minimizes LAD function *S*. Our problem (14) is convex since we are minimizing a convex LAD function over a set of real numbers, which gives us a unique global optimum. Consequently, it can be solved using standard statistical packages/software for quantile regression. The R package “*quantreg*” (Koenker, 2022) is used in our simulation study and real data analysis.

The ML1 condition (5), together with some additional regularity conditions, guarantees the consistency of the above ML1 estimator. That is, ${\hat{\Xi }}$ will converge to $\Xi ^*$ as the sample size *N* grows to infinity. This result is formalized in Theorem 1, with its proof given in the Supplementary Materials.

#### Theorem 1

Let $\Xi ^*=\{\beta ^*, (\sigma ^*)^2, \gamma _j^*, d_j^*, a_j^*, j=1, \ldots , J\}$ be the true model parameters, and $\Xi ^\dagger =\{\beta ^\dagger , (\sigma ^2)^\dagger , \gamma _j^\dagger , d_j^\dagger , a_j^\dagger , j=1, \ldots ,J\} = \Xi ^*(\gamma _1^*/a_1^*)$ be the true parameter values of the equivalent MIMIC model with constraint $\gamma _1^\dagger =0$. Assume this equivalent model satisfies the standard regularity conditions in Theorem 5.14 of van der Vaart (2000) that concerns the consistency of maximum likelihood estimation. Further, assume that the ML1 condition (5) holds. Then $\vert {\hat{\beta }} - \beta ^*\vert =o_P(1), {\vert \hat{\sigma }^2 -(\sigma ^2)^*\vert =o_P(1)}, \vert {\hat{\gamma }}_j - \gamma _j^*\vert =o_P(1)$, $\vert {\hat{a}}_j - a_j^*\vert =o_P(1), \text{ and } \vert {\hat{d}}_j - d_j^*\vert =o_P(1)$ as $N\rightarrow \infty$.

With a consistent point estimator, one can consistently select the true model, i.e., identifying the zeros and nonzeros in $\varvec{\gamma }^*$, using a hard-thresholding procedure (see, e.g. Meinshausen & Yu, 2009). As our focus is on the statistical inference of DIF parameters, we skip the details of the hard-thresholding procedure here. Once the final model is selected, it may be possible to verify the ML1 condition by checking whether the sufficient conditions in Corollary 1 hold for the selected model.

### Statistical Inference

The statistical inference of individual $\gamma _j$ parameters is of particular interest in the DIF analysis. With the proposed estimator, we can draw valid statistical inference on the DIF parameters $\gamma _j$.

Note that the uncertainty of ${\hat{\gamma }}_j$ is inherited from $\tilde{\Xi }$, where $\sqrt{N}(\tilde{\Xi }-\Xi ^\dagger )$ asymptotically follows a mean-zero multivariate normal distribution^1^ by the large-sample theory for maximum likelihood estimation; see Supplementary Materials for more details. We denote this multivariate normal distribution by $N({\textbf{0}}, \Sigma ^*)$, where a consistent estimator of $\Sigma ^*$, denoted by ${\hat{\Sigma }}_N$, can be obtained based on the marginal likelihood. We define a function

$$\begin{aligned} G_j(\Xi ) = \gamma _j - a_j \times \arg \min _{c} \sum _{l=1}^J \vert \gamma _l - a_lc\vert , \end{aligned}$$

where $\Xi = \{\beta , \sigma ^2, a_l, d_l, \gamma _l, l = 1, \ldots , J\}$. Note that the function $G_j$ maps an arbitrary parameter vector of the MIMC model to the $\gamma _j$ parameter of the equivalent ML1 parameter vector. To draw statistical inference, we need the distribution of

$$\begin{aligned} {\hat{\gamma }}_j - \gamma _j^* = G_j(\tilde{\Xi }) - G_j(\Xi ^\dagger ). \end{aligned}$$

By the asymptotic distribution of $\sqrt{N}(\tilde{\Xi }-\Xi ^\dagger )$, we know that the distribution of $G_j(\tilde{\Xi }) - G_j(\Xi ^\dagger )$ can be approximated by that of $G_j(\Xi ^\dagger + {\textbf{Z}}/\sqrt{N}) - G_j(\Xi ^\dagger )$, and the latter can be further approximated by $G_j(\tilde{\Xi }+ \textbf{Z}/\sqrt{N}) - G_j(\tilde{\Xi })$, where ${\textbf{Z}}$ follows a normal distribution $N({\textbf{0}}, {\hat{\Sigma }}_N)$. Therefore, although function $G_j$ does not have an analytic form, we can approximate the distribution of ${\hat{\gamma }}_j - \gamma _j^*$ by Monte Carlo simulation. We summarize this procedure in Algorithm 2. It can be viewed as a parametric bootstrap procedure (Davison and Hinkley, 1997; Zhang, 2018).

**Algorithm 2:**
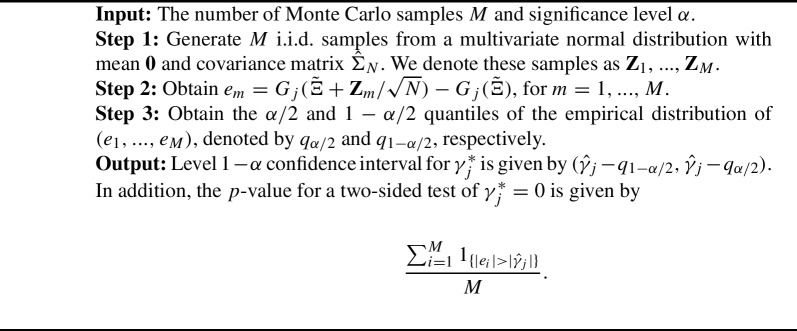
.

Algorithm 2 only involves sampling from a multivariate normal distribution and solving a convex optimization problem based on the LAD objective function, both of which are computationally efficient. The value of *M* is set to 10,000 in our simulation study and 50,000 in the real data example below.

The *P*-values can be used to control the type-I error rate, i.e., the probability of mistakenly detecting a non-DIF item as a DIF one. To control the item-specific type-I errors to be below a pre-specified threshold $\alpha$ (e.g., $\alpha = 0.05$), we detect the items for which the corresponding *P*-values are below $\alpha$. Besides the type-I error, we may also consider the false discovery rate (FDR) for DIF detection (Bauer et al., 2020) to account for multiple comparisons, where the FDR is defined as the expected ratio of the number of non-DIF items to the total number of detections. To control the FDR, the Benjamini–Hochberg (B-H) procedure (Benjamini and Hochberg, 1995) can be employed to the *P*-values. Other compound risks may also be considered, such as the familywise error rate.

## Related Works and Extensions

### Related Works

Many of the IRT-based DIF analyses (Thissen et al., 1986; Thissen, 1988; Thissen et al., 1993) require prior knowledge about a subset of DIF-free items, which are known as the anchor items. More precisely, we denote this known subset by *A*. Under the MIMIC model described above, it implies that the constraints $\gamma _j = 0$ are imposed for all $j \in A$ in the estimation. With these zero constraints, the $\gamma _j$ parameters cannot be freely transformed, and thus, the above MIMIC model becomes identifiable. Therefore, for each non-anchor item $j \notin A$, the hypothesis of $\gamma _j = 0$ can be tested, for example, by a likelihood ratio test. The DIF items can then be detected based on the statistical inference of these hypothesis tests.

The validity of the anchor-item-based analyses relies on the assumption that the anchor items are truly DIF-free. If the anchor set includes one or more DIF items, then the results can be misleading (Kopf et al., 2015b). To address the issue brought by the mis-specification of the anchor set, item purification methods (Candell and Drasgow, 1988; Clauser et al., 1993; Fidalgo et al., 2000; Wang and Yeh, 2003; Wang and Su, 2004; Wang et al., 2009; Kopf et al., 2015b, a) have been proposed that iteratively purify the anchor set. These methods conduct model selection using a stepwise procedure to select the anchor set, implicitly assuming that there exists a reasonably large set of DIF-free items. Then, DIF is assessed by hypothesis testing given the selected anchor set. This type of method also has several limitations. First, the model selection results may be sensitive to the choice of the initial set of anchor items and the specific stepwise procedure (e.g., forward or backward selection), which is a common issue with stepwise model selection procedures (e.g., stepwise variable selection for linear regression). Second, the model selection step has uncertainty. As a result, there is no guarantee that the selected anchor set is completely DIF-free, and furthermore, the post-selection statistical inference of items may not be valid in the sense that the type-I error may not be controlled at the targeted significance level.

Bechger and Maris (2015) and Yuan et al. (2021) proposed DIF detection methods based on the idea of differential item pair functioning. They considered a one-parameter logistic model setting, which corresponds to the case when $a_1 = \cdots = a_J$ in the current MIMIC model. Their idea is that the difference $\gamma _j - \gamma _{j'}$ is identifiable for any $j\ne j'$, though each individual $\gamma _j$ is not identifiable due to location indeterminacy. Bechger and Maris (2015) focused on testing $\gamma _j - \gamma _{j'} = 0$ for all item pairs, and Yuan et al. (2021) proposed data visualization methods and a Monte Carlo test to identify individual DIF items. However, they did not provide statistical inferences for the DIF effects of individual items. In particular, Yuan et al. (2021) constructed item-specific confidence intervals for the DIF effect parameters. However, their confidence intervals are constructed for an order statistic considering information from all the items and, thus, can only test the DIF effect of the item ranked in the *j*th place by their procedure. Moreover, their construction of confidence intervals requires a strong assumption that all the items are DIF-free, which does not hold in DIF detection problems. Our procedure does not require such an assumption.

Regularized estimation methods (Magis et al., 2015; Tutz and Schauberger, 2015; Huang, 2018; Belzak and Bauer, 2020; Bauer et al., 2020; Schauberger and Mair, 2020) have also been proposed for identifying the anchor items, which also implicitly assumes that many items are DIF-free. These methods do not require prior knowledge about anchor items and simultaneously select the DIF-free items and estimate the model parameters using a LASSO-type penalty (Tibshirani, 1996). Under the above MIMIC model, a regularized estimation procedure solves the following optimization problem,

15 $$\begin{aligned} {\hat{\Xi }}^\lambda = \arg \min _{\Xi } - \log L(\Xi ) + \lambda \sum _{j=1}^J \vert \gamma _j\vert , \end{aligned}$$

where $\lambda > 0$ is a tuning parameter that determines the sparsity level of the estimated $\gamma _j$ parameters. Generally speaking, a larger value of $\lambda$ leads to a more sparse vector $\hat{\varvec{\gamma }}^\lambda = ({{\hat{\gamma }}}_1^\lambda , \ldots , {\hat{\gamma }}_J^\lambda ).$ A regularization method (e.g. Belzak & Bauer, 2020) solves the optimization problem (15) for a sequence of $\lambda$ values and then selects the tuning parameter $\lambda$ based on the Bayesian information criterion (BIC; Schwarz, 1978). Let ${\hat{\lambda }}$ be the selected tuning parameter. Items for which ${{\hat{\gamma }}}_j^{{\hat{\lambda }}} \ne 0$ are classified as DIF items and the rest are classified as DIF-free items. While the regularization methods are computationally more stable than stepwise model selection in the item purification methods, they also suffer from some limitations. First, they involve solving non-smooth optimization problems like (15) for different tuning parameter values, which is not only computationally intensive but also requires tailored computation code that is not available in most statistical packages/software for DIF analysis. Second, these methods may be sensitive to the choice of the tuning parameter. Although methods and theories have been developed in the statistics literature to guide the selection of the tuning parameter, there is no consensus on how the tuning parameter should be chosen, leaving ambiguity in the application of these methods. Third, from the theoretical perspective, it is not clear whether these methods can guarantee model selection consistency. In particular, the model selection consistency of the LASSO procedure almost always requires a strong assumption called the irrepresentable condition (Zhao and Yu, 2006; van de Geer and Bühlmann, 2009). It is not clear when this assumption holds for the current problem. On the other hand, the proposed ML1 condition is much easier to understand and check, as discussed in Sect. 3.1. Finally, as a common issue of regularized estimation methods, obtaining valid statistical inference from these methods is not straightforward. That is, regularized estimation like (15) does not provide a valid *p*-value for testing the null hypothesis $\gamma _j = 0$. In fact, post-selection inference after regularized estimation was conducted in Bauer et al. (2020), where the type I error cannot be controlled at the targeted level under some simulation scenarios.

We notice that there is a connection between the proposed estimator and the regularized estimator (15). Note that ${\hat{\Xi }}$ is the one with the smallest $\sum _{j=1}^J \vert \gamma _j\vert$ among all equivalent estimators that maximize the likelihood function (3). When the solution path of (15) is smooth and the solution to the ML1 problem (14) is unique, it is easy to see that ${\hat{\Xi }}$ is the limit of ${\hat{\Xi }}^{\lambda }$ when the positive tuning parameter $\lambda$ converges to zero. In other words, the proposed estimator can be viewed as a limiting version of the LASSO estimator (15). According to Theorem 1, this limiting version of the LASSO estimator is statistically consistent under the ML1 condition and some reasonable regularity conditions.

We clarify that the proposed method may not always outperform other methods in terms of accuracy in classifying items, such as the LASSO procedure. From the simulation results in Sect. 5, we see that the proposed method and the LASSO procedure have similar accuracy in item classification when the DIF parameters are large. The key advantage of the proposed method is that the proposed one provides valid statistical inference (e.g., *P*-values) when anchor items are not available. The inference results allow us to tackle the uncertainty in the decisions of DIF detection, which can be useful in many applications of DIF analysis where high-stake decisions need to be made.

### Extensions

While we focus on the two-group setting and uniform-DIF (i.e., only the intercepts depend on the groups) in the previous discussion, the proposed framework is very general that can be easily generalised to other settings. In what follows, we discuss the ML1 condition under different settings. The proposed methods for point estimation and statistical inference can be extended accordingly.

**Non-uniform DIF.** Under the 2PL measurement model, non-uniform DIF happens when the discrimination parameter also differs across groups. To model non-uniform DIF, we extend the current measurement model (2) to

16 $$\begin{aligned} P(Y_{ij} = 1\vert \theta _i = \theta , x_i) = \frac{\exp (a_j\exp (\zeta _j x_i)\theta + d_j + \gamma _j x_i)}{1+\exp (a_j\exp (\zeta _j x_i)\theta + d_j + \gamma _j x_i)}, \end{aligned}$$

while keeping the structural model the same as in Sect. 2.2. This extended model has both location and scale indeterminacies. Let $\Xi ^* = \{\beta ^*, (\sigma ^*)^2, a_j^*, d_j^*, \zeta _j^*, \gamma _j^*, j = 1, \ldots , J\}$ be a set of parameters for the true model. Then, a set of parameters yields the same data distribution as the true model if there exist constants *m* and *c* such that $\Xi ^*(m,c) = \{(\beta ^*-c)\times \exp (-m), \exp (-2\,m)\times (\sigma ^*)^2, a_j^*, d_j^*, \zeta _j^* + m, \gamma _j^* - ca_j^*\exp (\zeta _j^*), j = 1, \ldots , J\}$. Note that an item *j* is DIF-free if $\zeta _j = \gamma _j =0$. Under the same spirit as the ML1 condition (5), we may assume the true model parameters $\Xi ^*$ to satisfy

$$\begin{aligned} {\sum _{j=1}^J \vert \zeta _j^* \vert< \sum _{j=1}^J \vert \zeta _j^* +m \vert }, \text{ and } \sum _{j=1}^J\vert \gamma _j^*\vert < \sum _{j=1}^J\vert \gamma _j^* + ca_j^*\exp (\zeta _j^*)\vert \end{aligned}$$

when $m\ne 0$ and $c\ne 0$. These conditions tend to be satisfied when the proportion of DIF-free items is sufficiently large.

**Multi-group setting.** There may be more than two groups in some DIF applications. Suppose that there are $K+1$ groups—one reference group and *K* focal groups. Let $x_i \in \{0, \ldots , K\}$ indicate the group membership.

For simplicity, we focus on the uniform DIF setting. Then, the measurement model becomes:

17 $$\begin{aligned} P(Y_{ij} = 1\vert \theta _i = \theta , x_i =k) = \frac{\exp (a_j \theta + d_j + \gamma _{jk} )}{1+\exp (a_j \theta + d_j + \gamma _{jk})}, k = 1, \ldots , K, \end{aligned}$$

and

18 $$\begin{aligned} P(Y_{ij} = 1\vert \theta _i = \theta , x_i =0) = \frac{\exp (a_j \theta + d_j )}{1+\exp (a_j \theta + d_j ) }. \end{aligned}$$

The structural model becomes $\theta _i \vert x_i=k \sim N(\beta _k, \sigma _k^2), k = 1, \ldots , K,$ and $\theta _i \vert x_i=0 \sim N(0, 1)$. Under this model, an item *j* is DIF-free if $\gamma _{jk} = 0$ for all *k*. The location indeterminacy under this model leads to the following ML1 condition for identifying the true model parameters $\Xi ^* = \{\beta ^*_k, (\sigma _k^*)^2, a_j^*, d_j^*, \gamma _{jk}^*, k = 1, \ldots , K, j = 1, \ldots , J,\}$:

$$\begin{aligned} \sum _{j=1}^{J} \vert \gamma _{jk}^* \vert < \sum _{j=1}^{J} \vert \gamma _{jk}^* -a_j^* c_k\vert , \end{aligned}$$

for $$c_k\ne 0$$, $$k = 1, \ldots , K$$.

We note that this ML1 condition for the multi-group setting allows the majority of the items to be DIF items as long as the vector $(\gamma _{1k}^*, \ldots , \gamma _{Jk}^*)^\top$ is sufficiently sparse for each focal group. Similar to the discussion in Sect. 3.1, in the special case of the one-parameter logistic model, the ML1 condition is guaranteed to hold if $\sum _{j=1}^J I(\gamma _{jk}^* = 0) > J/2$, for all *k*. Note that the set of items satisfying $\gamma _{jk}^* = 0$ can vary across focal groups.

**Continuous covariates.** In some applications, DIF might be caused by continuous covariates, such as age. Suppose that we have *K* continuous covariates ${\textbf{x}}_i = (x_{i1}, \ldots , x_{iK})^\top$, rather than discrete groups. Then, we may consider the following measurement model:

19 $$\begin{aligned} P(Y_{ij} = 1\vert \theta _i = \theta , {\textbf{x}}_i) = \frac{\exp (a_j \theta + d_j +\varvec{\gamma }_j^\top {\textbf{x}}_{i} )}{1+\exp (a_j \theta + d_j +\varvec{\gamma }_j^\top {\textbf{x}}_{i})}, \end{aligned}$$

where $\varvec{\gamma }_j = (\gamma _{j1}, \ldots , \gamma _{jK})^\top$ be the corresponding DIF parameters. We may assume the structural model takes a homoscedastic latent regression form $\theta \vert {\textbf{x}}_i \sim N(\varvec{\beta }{\textbf{x}}_i,1)$, where the variance is fixed to 1 to avoid scale indeterminacy.^2^ Under this MIMIC model, an item *j* is DIF-free if $\gamma _{jk} = 0$ for all *k*. The location indeterminacy under this model leads to the following ML1 condition for identifying the true model parameters $\Xi ^* = \{\beta ^*_k, a_j^*, d_j^*, \gamma _{jk}^*, k = 1, \ldots , K, j = 1, \ldots , J,\}$:

$$\begin{aligned} \sum _{j=1}^{J} \vert \gamma _{jk}^* \vert < \sum _{j=1}^{J} \vert \gamma _{jk}^* -a_j^* c_k\vert , \end{aligned}$$

for $$c_k\ne 0$$, $$k = 1, \ldots , K$$.

We note that this ML1 condition is similar to that under the multi-group setting. This is because the multi-group setting can be written in a very similar form as the current MIMIC model (by representing the groups using a covariate vector with dummy variables), except that the structural model under the multi-group setting allows heteroscedasticity. We also note that the current model assumes that a DIF effect is a linear combination of the covariates, which may seem inflexible, especially when comparing with the tree-based methods (Strobl et al., 2015; Tutz and Berger, 2016; Bollmann et al., 2018). However, we note that one can always move beyond the linearity by including transformations of the raw covariates (e.g., using spline basis) into the covariate vector and increasing the dimension of the DIF parameter vector $\varvec{\gamma }_j$ simultaneously.

**Ordinal response data.** Finally, we note that the proposed method can be extended to IRT models for other types of response data. To elaborate, we consider the generalized partial credit model (GPCM) (Muraki, 1992) for ordinal response data as an example. For simplicity, we focus on the two-group setting (i.e., $x_i \in \{0,1\}$) and uniform DIF. Let $\{0, 1, \ldots , m_j\}$ be the ordered categories of item *j*. Then, the measurement model becomes:

$$\begin{aligned} \frac{P(Y_{ij} = k\vert \theta _i = \theta , x_i)}{P(Y_{ij} = k-1\vert \theta _i = \theta , x_i)} = {\exp (a_j\theta + d_{jk} + \gamma _{jk}x_i)}, k = 1, \ldots ,m_j, \end{aligned}$$

where the DIF parameters $\gamma _{jk}$ depend on both the item and the category. We keep the structural model the same as in Sect. 2.2. Under this model, an item *j* is DIF-free if $\gamma _{jk} = 0$ for all *k*. The location indeterminacy under this model leads to the following ML1 condition for identifying the true model parameters $\Xi ^* = \{\beta ^*, (\sigma ^*)^2, a_j^*, d_j^*, \gamma _{jk}^*, k = 1, \ldots , m_j, j = 1, \ldots , J\}$:

$$\begin{aligned} \sum _{j=1}^{J}\sum _{k=1}^{m_j} \vert \gamma _{jk}^* \vert < \sum _{j=1}^{J}\sum _{k=1}^{m_j} \vert \gamma _{jk}^* -a_j^* c\vert , \end{aligned}$$

for all $$c\ne 0$$.

## Simulation Study

This section conducts simulation studies to evaluate the performance of the proposed method and compare it with the likelihood ratio test (LRT) method (Thissen, 1988) and the LASSO method (Bauer et al., 2020). Note that the LRT method requires a known anchor item set. Correctly specified anchor item sets with different sizes will be considered when applying the LRT method.

In the simulation, we set the number of items $J = 25$ and consider two settings for the sample sizes, $N = 500$, and 1000. The parameters of the true model are set as follows. First, the discrimination parameters are set between 1 and 2, and we consider two sets of easiness parameters with one small $d_j$ set between $-1$ and 1 and another large $d_j$ set between $-2$ and 2, respectively. Their true values are given in Table 1. The observations are split into groups of equal sizes, indicated by $x_i = 0$, and 1. The parameter $\beta$ in the structural model is set to 0.5 and the parameter $\sigma$ is set to 0.5, so that the latent trait distribution is standard normal *N*(0, 1) and $N(0.5, 0.5^2)$ for the reference and focal groups, respectively. We consider six settings for the DIF parameters, three settings with DIF item proportions from high to low at smaller absolute DIF parameter values, and the other three with DIF item proportions from high to low at larger absolute DIF parameter values. Specifically, at smaller and larger absolute DIF parameter values, the three settings contain 5, 10 and 14 DIF items out of 25 items for low, medium and high DIF proportions, respectively. Their true values are given in Table 1. For all sets of the DIF parameters, the ML1 condition is satisfied. The combinations of settings for the sample sizes and DIF parameters lead to 24 settings in total. For each setting, 100 independent datasets are generated.

We first evaluate the accuracy of the proposed estimator ${\hat{\Xi }}$ given by Algorithm 1. Table 2 shows the mean-squared errors (MSE) for $\beta$ and $\sigma$ and the average MSEs for $a_j$s, $d_j$s, and $\gamma _j$s that are obtained by averaging the corresponding MSEs over the *J* items. As we can see, these MSEs and average MSEs are small in magnitude and decrease as the sample size of individuals *N* increases under each setting. This observation aligns with our consistency result in Theorem 1.

We then compare the proposed method and the LRT method in terms of their performances on statistical inference. Specifically, we focus on whether FDR can be controlled when applying the B-H procedure to the *P*-values obtained from the two methods. The comparison results are given in Table 3. As we can see, FDR is controlled to be below the targeted level for the proposed method and the LRT method with 1, 5, and 10 anchor items under all settings.

**Fig. 3:**
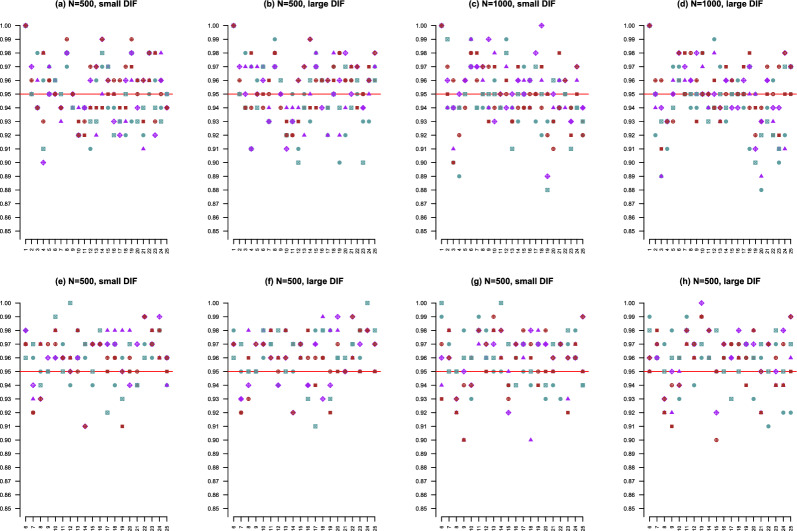
Scatter plots of the coverage rates of the 95% confidence intervals for $\gamma _j^*$’s. x-axes and y-axes are labelled with item numbers and coverage rates, respectively. Panels **a**–**d** correspond to our proposed method, and panels **e**–**h** correspond to the Wald intervals constructed with five anchor items. Blue solid circle corresponds to small $d_j$ with high proportion DIF items. Purple solid triangle corresponds to small $d_j$ with medium proportion DIF items. Red solid square corresponds to small $d_j$ with low proportion DIF items. Blue square cross corresponds to large $d_j$ with high proportion DIF items. Purple diamond plus corresponds to large $d_j$ with medium proportion DIF items. Red circle plus corresponds to large $d_j$ with low proportion DIF items.

When anchor items are known, the standard error can be computed for each estimated $\gamma _j$, and thus, the corresponding Wald interval can be constructed. We compare the coverage rates of the confidence intervals given by Algorithm 2 and the Wald intervals that are based on five anchor items. The results are shown in Fig. 3. We see that the coverage rates from both methods are comparable across all settings and are close to the 95% targeted level. Note that these coverage rates are calculated based on only 100 replicated datasets, which may be slightly affected by the Monte Carlo errors.

Finally, we compare the detection power of different methods based on the receiver operating characteristic (ROC) curves. For a given method, a ROC curve is constructed by plotting the true-positive rate (TPR) against the false-positive rate (FPR) at different threshold settings. More specifically, ROC curves are constructed for the LASSO methods by varying the corresponding tuning parameters $\lambda$ from 0.02 to 0.2 where the optimal $\lambda$ is selected using the BIC. ROC curves are also constructed by the LRT method with 1, 5, and 10 anchor items, respectively. Note that for the LRT method, the TPR and FPR are calculated based on the non-anchor items. For each method, an average ROC curve is obtained based on the 100 replications, for which the area under the ROC curve (AUC) is calculated. A larger AUC value indicates better detection power. The AUC values for different methods across our simulation settings are given in Table 4. According to the AUC values, the proposed procedure, that is, the *P*-value-based method from Algorithm 2, performs better than the rest. That is, without knowing any anchor items, the proposed procedure performs better than the LRT method that knows 1 or 5 anchor items and has similar performance as the LRT method that knows 10 anchor items under some settings with large DIF or large sample size *N*. The superior performance of the proposed procedures is brought by the use of the ML1 condition, which identifies the model parameters using information from all the items. Based on the AUC values, we also see that the LASSO procedure performs similarly to the proposed procedures under some of the large DIF settings, but is less accurate under the small DIF settings.

**Table 1:** Discrimination, easiness and DIF parameter values used in the simulation studies.

Item number	$a_j$	$d_j$		$\gamma _j$ (Small DIF)			$\gamma _j$ (Large DIF)		
		Small $d_j$	Large $d_j$	High	Medium	Low	High	Medium	Low
1	1.30	0.80	0.80	0.00	0.00	0.00	0.00	0.00	0.00
2	1.40	0.20	$-$ 0.40	0.00	0.00	0.00	0.00	0.00	0.00
3	1.50	$-$ 0.40	$-$ 1.20	0.00	0.00	0.00	0.00	0.00	0.00
4	1.70	$-$ 1.00	$-$ 2.00	0.00	0.00	0.00	0.00	0.00	0.00
5	1.60	1.00	2.00	0.00	0.00	0.00	0.00	0.00	0.00
6	1.30	0.80	0.80	0.00	0.00	0.00	0.00	0.00	0.00
7	1.40	0.20	$-$ 0.40	0.00	0.00	0.00	0.00	0.00	0.00
8	1.50	$-$ 0.40	$-$ 1.20	0.00	0.00	0.00	0.00	0.00	0.00
9	1.70	$-$ 1.00	$-$ 2.00	0.00	0.00	0.00	0.00	0.00	0.00
10	1.60	1.00	2.00	0.00	0.00	0.00	0.00	0.00	0.00
11	1.30	0.80	0.80	0.00	0.00	0.00	0.00	0.00	0.00
12	1.40	0.20	$-$ 0.40	$-$ 0.60	0.00	0.00	$-$ 1.20	0.00	0.00
13	1.50	$-$ 0.40	$-$ 1.20	0.60	0.00	0.00	1.20	0.00	0.00
14	1.70	$-$ 1.00	$-$ 2.00	$-$ 0.65	0.00	0.00	$-$ 1.30	0.00	0.00
15	1.60	1.00	2.00	0.70	0.00	0.00	1.40	0.00	0.00
16	1.30	0.80	0.80	$-$ 0.60	$-$ 0.60	0.00	$-$ 1.20	$-$ 1.20	0.00
17	1.40	0.20	$-$ 0.40	0.60	0.60	0.00	1.20	1.20	0.00
18	1.50	$-$ 0.40	$-$ 1.20	$-$ 0.65	$-$ 0.65	0.00	$-$ 1.30	$-$ 1.30	0.00
19	1.70	$-$ 1.00	$-$ 2.00	0.70	0.70	0.00	1.40	1.40	0.00
20	1.60	1.00	2.00	0.65	0.65	0.00	1.30	1.30	0.00
21	1.30	0.80	0.80	$-$ 0.60	$-$ 0.60	$-$ 0.60	$-$ 1.20	$-$ 1.20	$-$ 1.20
22	1.40	0.20	$-$ 0.40	0.60	0.60	0.60	1.20	1.20	1.20
23	1.50	$-$ 0.40	$-$ 1.20	$-$ 0.65	$-$ 0.65	$-$ 0.65	$-$ 1.30	$-$ 1.30	$-$ 1.30
24	1.70	$-$ 1.00	$-$ 2.00	0.70	0.70	0.70	1.40	1.40	1.40
25	1.60	1.00	2.00	0.65	0.65	0.65	1.30	1.30	1.30

**Table 2:** Average mean-squared errors of the estimated parameters in the simulation studies.

			Small DIF			Large DIF		
			High	Medium	Low	High	Medium	Low
$N = 500$	Small $d_j$	$\varvec{a}$	0.0482	0.0485	0.0485	0.0502	0.0490	0.0486
		$\varvec{d}$	0.0316	0.0317	0.0318	0.0317	0.0317	0.0316
		$\varvec{\gamma }$	0.0614	0.0612	0.0609	0.0670	0.0650	0.0623
		${\beta }$	0.0010	0.0010	0.0010	0.0011	0.0011	0.0010
		${\sigma }$	0.0016	0.0017	0.0016	0.0017	0.0017	0.0018
	Large $d_j$	$\varvec{a}$	0.0562	0.0552	0.0552	0.0589	0.0575	0.0560
		$\varvec{d}$	0.0467	0.0467	0.0470	0.0475	0.0476	0.0476
		$\varvec{\gamma }$	0.0873	0.0854	0.0834	0.1089	0.1009	0.0903
		${\beta }$	0.0013	0.0012	0.0012	0.0011	0.0012	0.0013
		${\sigma }$	0.0014	0.0014	0.0015	0.0015	0.0015	0.0015
$N = 1000$	Small $d_j$	$\varvec{a}$	0.0227	0.0222	0.0222	0.0223	0.0221	0.0222
		$\varvec{d}$	0.0145	0.0145	0.0145	0.0145	0.0145	0.0145
		$\varvec{\gamma }$	0.0291	0.0289	0.0287	0.0335	0.0320	0.0298
		${\beta }$	0.0004	0.0004	0.0004	0.0005	0.0005	0.0004
		${\sigma }$	0.0004	0.0005	0.0005	0.0005	0.0005	0.0005
	Large $d_j$	$\varvec{a}$	0.0263	0.0261	0.0262	0.0270	0.0267	0.0264
		$\varvec{d}$	0.0223	0.0224	0.0225	0.0227	0.0224	0.0226
		$\varvec{\gamma }$	0.0412	0.0401	0.0392	0.0500	0.0461	0.0418
		${\beta }$	0.0005	0.0005	0.0005	0.0006	0.0005	0.0005
		${\sigma }$	0.0005	0.0005	0.0005	0.0006	0.0005	0.0005

Mean-squared errors are first evaluated by averaging out of 100 replications and then averaged across 25 items to obtain the average mean-squared errors for $\varvec{a}$, $\varvec{d}$ and $\varvec{\gamma }$. The mean-squared errors for $\beta$ and $\sigma$ are presented

**Table 3:** Comparison of the FDR of the proposed *P*-value based method and the LRT method with 1, 5 and 10 anchor items, respectively, at the FDR control of 5%. The values are averaged out of 100 replications.

			Small DIF			Large DIF		
			High	Medium	Low	High	Medium	Low
$N = 500$	Small $d_j$	proposed	0.0167	0.0255	0.0298	0.0192	0.0213	0.0319
		LRT 1	0.0089	0.0071	0.0137	0.0119	0.0148	0.0233
		LRT 5	0.0071	0.0181	0.0267	0.0122	0.0195	0.0394
		LRT 10	0.0033	0.0148	0.0283	0.0027	0.0154	0.0329
	Large $d_j$	proposed	0.0240	0.0222	0.0323	0.0231	0.0249	0.0404
		LRT 1	0.0164	0.0212	0.0267	0.0152	0.0216	0.0280
		LRT 5	0.0124	0.0221	0.0308	0.0128	0.0215	0.0246
		LRT 10	0.0031	0.0219	0.0237	0.0029	0.0159	0.0408
$N = 1000$	Small $d_j$	proposed	0.0238	0.0277	0.0349	0.0229	0.0269	0.0425
		LRT 1	0.0087	0.0083	0.0152	0.0083	0.0131	0.0170
		LRT 5	0.0100	0.0217	0.0327	0.0087	0.0218	0.0341
		LRT 10	0.0021	0.0191	0.0389	0.0020	0.0162	0.0408
	Large $d_j$	proposed	0.0217	0.0302	0.0390	0.0227	0.0333	0.0444
		LRT 1	0.0165	0.0166	0.0248	0.0172	0.0193	0.0237
		LRT 5	0.0114	0.0155	0.0249	0.0100	0.0162	0.0250
		LRT 10	0.0007	0.0062	0.0218	0.0013	0.0079	0.0260

**Table 4:** Comparison of AUC of the proposed *P*-value-based method, the LASSO method and the LRT method with 1, 5 and 10 anchor items, respectively.

			Small DIF			Large DIF		
			High	Medium	Low	High	Medium	Low
$N = 500$	Small $d_j$	proposed	0.936	0.933	0.942	0.996	0.997	0.998
		LASSO	0.802	0.805	0.789	0.992	0.991	0.987
		LRT 1	0.861	0.853	0.867	0.982	0.984	0.982
		LRT 5	0.915	0.917	0.920	0.992	0.991	0.988
		LRT 10	0.929	0.919	0.922	0.989	0.995	0.989
	Large $d_j$	proposed	0.910	0.915	0.917	0.986	0.988	0.990
		LASSO	0.685	0.672	0.670	0.920	0.938	0.936
		LRT 1	0.823	0.800	0.826	0.966	0.966	0.969
		LRT 5	0.884	0.878	0.881	0.980	0.980	0.978
		LRT 10	0.897	0.875	0.884	0.983	0.975	0.977
$N = 1000$	Small $d_j$	proposed	0.984	0.986	0.987	1.000	1.000	1.000
		LASSO	0.815	0.818	0.817	0.995	0.995	0.993
		LRT 1	0.965	0.968	0.960	0.997	0.997	0.994
		LRT 5	0.979	0.975	0.976	0.990	0.990	0.990
		LRT 10	0.985	0.966	0.977	0.995	0.984	0.988
	Large $d_j$	proposed	0.964	0.964	0.965	0.997	0.998	0.998
		LASSO	0.685	0.673	0.667	0.937	0.953	0.947
		LRT 1	0.944	0.942	0.941	0.989	0.995	0.992
		LRT 5	0.962	0.961	0.962	0.990	0.993	0.992
		LRT 10	0.972	0.953	0.962	1.000	0.998	0.992

## Application to EPQ-R Data

DIF methods have been commonly used for assessing the measurement invariance of personality tests (e.g., Escorial & Navas, 2007, Millsap, 2012, Thissen et al., 1986). In this section, we apply the proposed method to the Eysenck Personality Questionnaire-Revised (EPQ-R, Eysenck et al. 1985), a personality test that has been intensively studied and received applications worldwide (Fetvadjiev and van de Vijver, 2015). The EPQ-R has three scales that measure the Psychoticism (P), Neuroticism (N) and Extraversion (E) personality traits, respectively. We analyse the long forms of the three personality scales that consist of 32, 24, and 23 items, respectively. Each item has binary responses of “yes” and “no” that are indicated by 1 and 0, respectively. This analysis is based on data from an EPQ-R study collected from 1432 participants in the UK. Among these participants, 823 are females, and 609 are males. Females and males are indicated by $x_i = 0$ and 1, respectively. We study the DIF caused by gender. The three scales are analysed separately using the proposed methods.

**Fig. 4:**
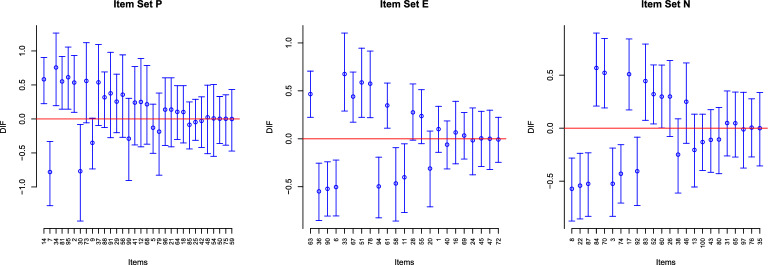
Plots of 95% confidence intervals for the DIF parameters $\gamma _j^{*'}s$ on scale P, N, and E data sets. The red horizontal lines denote $\gamma =0$. Items are arranged according to the increasing *P*-values.

The results are shown through Tables 5–7, and Fig. 4. Specifically, Tables  5–7 present the *P*-values from the proposed method for testing $\gamma _j = 0$ and the detection results for the P, E, N scales, respectively. For each table, the items are ordered by the *P*-values in increasing order. The items indicated by “F” are the ones detected by the B-H procedure with FDR level 0.05, and those indicated by “L” are the ones detected by LASSO method whose tuning parameter $\lambda$ is chosen by BIC. The item IDs are consistent with those in Appendix 1 of Eysenck et al. (1985), where the item descriptions are given. The three panels of Fig. 4 further give the point estimate and confidence interval for each $\gamma _j$ parameter, for the three scales, respectively. Under the current model parameterization, a positive DIF parameter means that a male participant is more likely to answer “yes” to the item than a female participant, given that they have the same personality trait level. We note that the absolute values of ${\hat{\gamma }}_j$ are all below 1, suggesting that there are no items with very large gender-related DIF effects.

From Tables 5–7, we see that all three scales have some items whose *P*-values are close to zero, suggesting that gender DIF may exist across the three scales. The DIF items selected by the B-H procedure at the 5% FDR level seem sensible. In what follows, we give some examples. For the P scale, the top four items are selected. These items are “14. Do you dislike people who don’t know how to behave themselves?”, “7. Would being in debt worry you?”, “34. Do you have enemies who want to harm you?” and “81. Do you generally ‘look before you leap’?”, with the DIF effect of item 7 being negative while those of the rest being positive. The discovery of items 14, 7 and 34 is consistent with the personality literature, where previous research has found that women are more gregarious and trusting than men while men tend to be more risk-taking (Costa et al., 2001; Feingold, 1994). It is unclear from previous research why item 81 has a positive DIF effect. We conjecture that it is due to sociocultural influences. This result is consistent with that of another P-scale item “2. Do you stop to think things over before doing anything?" whose statement is similar to item 81. Although not selected by the B-H procedure, the estimated DIF effect of this item is also positive, and its 95% confidence interval does not include zero.

For the E scale, eleven items are selected by the B-H procedure. Here, we discuss the top five items, including “63. Do you nearly always have a ‘ready answer’ when people talk to you?”, “36. Do you have many friends?”, “90. Do you like plenty of bustle and excitement around you?”, “6. Are you a talkative person?" and “33. Do you prefer reading to meeting people?", where items 63 and 33 have positive DIF effects while the rest three have negative DIF effects. The discovery of these items is not surprising. The DIF effects of items 36, 90, 6 and 33 are consistent with previous observations that women are more motivated to involve in social activities and tend to have more interconnected and affiliative social groups (Cross and Madson, 1997), which may be explained by the theory of self-construals (Markus and Kitayama, 1991). The DIF effect of item 63 is consistent with the previous findings that men tend to score higher on assertiveness (Costa et al., 2001; Feingold, 1994; Weisberg et al., 2011).

For the N scale, ten items are selected by the B-H procedure. Again, we discuss the top five items, including “8. Do you ever feel ‘just miserable’ for no reason?”, “22. Are your feelings easily hurt?”, “87. Are you easily hurt when people find fault with you or the work you do?”, “84. Do you often feel lonely?" and “70. Do you often feel life is very dull?", where items 8, 22 and 87 have negative DIF effects and items 84 and 70 have positive DIF effects. The discovery of items 8, 22, and 87 is consistent with the fact that women tend to score higher in tender-mindedness (Costa et al., 2001; Feingold, 1994). The positive DIF effects of items 84 and 70 may again be explained by the theory of self-construals (Markus and Kitayama, 1991).

From Tables 5–7, we see that the selection based on the B-H procedure with FDR level 0.05 and that based on the LASSO procedure are quite consistent but do not exactly match. For the P-scale, the two procedures agree on four DIF detections, while the LASSO procedure additionally identifies four DIF items. For the E scale, they agree on six DIF detections, while the B-H procedure additionally identifies five items and the Lasso procedure additionally identifies one. Finally, for the N scale, the number of common detections is eight. Besides that, there are two items uniquely identified by the B-H procedure and four items uniquely identified by the Lasso procedure. Since the two procedures have different objectives (controlling FDR versus consistent model selection), it is not surprising that their results are not exactly the same. A consensus between the two methods suggests strong evidence, and thus, these common detections should draw our attention and be investigated first. For example, the content of the DIF items may be reviewed by experts, and new data may be collected to test these DIF effects through a confirmatory analysis. When there are enough resources, the items identified by one of the methods should also be investigated.

**Table 5:** *P*-values for testing $\gamma _j^*=0$ for items in P scale.

Item	14 FL	7 FL	34 FL	81 FL	95 L	2 L	30	73
*P*-value	0.0014	0.0015	0.0057	0.0061	0.0104	0.0140	0.0364	0.0619
Item	9L	37 L	88	91	29	56	99	41
*P*-value	0.0625	0.0681	0.1235	0.2217	0.2304	0.2442	0.3389	0.3780
Item	12	68	5	79	96	21	64	18
*P*-value	0.4389	0.4557	0.4567	0.5187	0.5515	0.5529	0.5819	0.5888
Item	85	25	42	48	54	50	75	59
*P*-value	0.6080	0.7527	0.8441	0.8787	0.9447	0.9528	0.9559	0.9616

Note that the items are ordered in increasing *P*-values. Items selected by the B-H procedure with FDR control at 5% and the LASSO method are identified using “F” and “L”, respectively, following the item numbers.

**Table 6:** *P*-values for testing $\gamma _j^*=0$ for items in E scale.

Item	63 FL	36 F	90 F	6 F	33 FL	67 FL	51 FL	78 FL
*P*-value	0.0000	0.0004	0.0006	0.0011	0.0013	0.0013	0.0016	0.0019
Item	94 F	61 FL	58 F	11	28L	55	20	1
*P*-value	0.0031	0.0051	0.0199	0.0310	0.0644	0.0958	0.1278	0.4073
Item	40	16	69	24	45	47	72	
*P*-value	0.6185	0.6439	0.7819	0.8371	0.9291	0.9364	0.9391	

Note that the items are ordered in increasing *P*-values. Items selected by the B-H procedure with FDR control at 5% and the LASSO method are identified using “F” and “L”, respectively, following the item numbers.

**Table 7:** *P*-values for testing $\gamma _j^*=0$ for items in N scale.

Item	8 FL	22 FL	87 FL	84 FL	70 FL	3 F	74 FL	17 FL
*P*-value	0.0004	0.0006	0.0007	0.0014	0.0016	0.0026	0.0026	0.0037
Item	92 F	83 FL	52 L	60 L	26 L	38	46 L	13
*P*-value	0.0130	0.0152	0.0264	0.0487	0.0994	0.1553	0.1856	0.2337
Item	100	43	80	31	65	97	76	35
*P*-value	0.3365	0.4417	0.4694	0.7116	0.7376	0.9220	0.9531	0.9550

Note that the items are ordered in increasing *P*-values. Items selected by the B-H procedure with FDR control at 5% and the LASSO method are identified using “F” and “L”, respectively, following the item numbers.

## Discussion

This paper proposes a new method for DIF analysis under a MIMIC model framework. It can accurately estimate the DIF effects of individual items without requiring prior knowledge about an anchor item set and can also provide valid *P*-values. The *P*-values can be used for the detection of DIF items and controlling the uncertainty in the decisions. According to our simulation results, the proposed *P*-value-based procedure has comparable performance in terms of classifying DIF and non-DIF items, comparing with the LASSO method of Belzak and Bauer (2020). In addition, the *P*-value-based methods accurately control the item-specific type-I errors and the FDR. Finally, the proposed method is applied to the three scales of the Eysenck Personality Questionnaire-Revised to study gender-related DIF. For each of the three long forms of the P, N, and E scales, around 10 items are detected by the proposed procedures as potential DIF items. The psychological mechanism of these DIF effects is worth further investigation. While the paper focuses on the two-group setting and uniform DIF, extensions to more complex settings are discussed in Sect. 4, including non-uniform DIF, multi-group, and continuous covariate, and ordinal response settings. The R functions for performing the proposed procedures are available from “https://github.com/Austinlccvic/DIF-Statistical-Inference-and-Detection-without-Knowing-Anchoring-Items”.

The proposed method has several advantages over the LASSO method. First, the proposed method does not require a tuning parameter to estimate the model parameters, while the LASSO method involves choosing the tuning parameter for the regularization term. Thus, the proposed method is more straightforward to use for practitioners. Second, we do not need to solve optimization problems that involve maximizing a regularized likelihood function under different tuning parameter choices. Therefore, the proposed method is computationally less intensive since the optimization involving a regularized likelihood function is non-trivial due to both the integral with respect to the latent variables and the non-smooth penalty term. Finally, the proposed method provides valid statistical inference, which is more difficult for the LASSO method due to the uncertainty associated with the model selection step. With the obtained *P*-values, the proposed approach can detect the DIF items with controlled type-I error or FDR.

The current work has some limitations, which offer opportunities for future research. First, we note that the proposed method relies heavily on the ML1 condition, which holds when the proportion of DIF-free items is sufficiently high. While it may be sensible to make this assumption in many applications, there may also be applications where the proportion of DIF items is high, in which case the ML1 condition may fail to hold. For example, as discussed earlier, the ML1 condition fails under a one-parameter logistic model when the proportion of DIF items is more than 50%. Methods remain to be developed under such settings. One possible idea is to replace the $L_1$ norm in the ML1 condition with an $L_p$ norm for some $p \in (0,1)$. The $L_p$ norm better approximates the $L_0$ norm; thus, the corresponding condition is more likely to hold under a less sparse setting. However, the computation becomes more challenging when using the $L_p$ norm, as the transformation in Step 2 of Algorithm 1 is no longer a convex optimization problem. Second, as is true for all simulation studies, we cannot examine all possible conditions that might occur in applied settings. Additional simulation studies will be conducted in future research to understand the performance of the proposed method better. In particular, sample sizes, item sizes, group sizes and distribution of the DIF items can be varied and tested. Third, the robustness of the proposed method remains to be studied when the ML1 condition is slightly violated. That is, it might be the case that $\varvec{\gamma }^*$ is approximately sparse—a high proportion of its entries are close to but not exactly zero. Given the continuity of the LAD optimisation problem (14), we expect that the proposed method can still effectively detect the items with large values of $|\gamma _j^*|$. However, in the meantime, we expect the *P*-values and confidence intervals to be slightly compromised due to the bias brought by the violation of the ML1 condition. A sensitivity analysis is needed to investigate the consequences. Fourth, although the extensions to several more complex settings are discussed in Sect. 4, these procedures remain to be implemented and assessed by simulation studies. Finally, the current work focuses on the type-I error and FDR as error metrics that concern falsely detecting non-DIF items as DIF items. In many applications of measurement invariance, it may also be of interest to consider an error metric that concerns the false detection of DIF items as DIF-free. Suitable error metrics, as well as methods for controlling such error metrics, remain to be proposed.

Although we focus on the DIF detection problem, the proposed method is also closely related to the problem of linking multiple groups’ test results in the violation of measurement invariance (Asparouhov and Muthén, 2014; Haberman, 2009; Robitzsch, 2020). Robitzsch (2020) proposed a linking approach based on an $L_p$ loss function, which is similar in spirit to the proposed method but focuses on linking multiple groups rather than DIF detection. We believe the proposed method can easily adapt to the linking problem to provide consistent parameter estimation and valid statistical inference. This problem is left for future investigation.

### Supplementary Information

Below is the link to the electronic supplementary material.

Supplementary file 1 (pdf 173 KB)

## Footnotes

1 Note that this is a degenerated multivariate normal distribution since $\tilde{\gamma }_1 = \gamma _1^\dagger =0$.

2 We note that the homoscedastic assumption is commonly adopted in structural equation models. It is possible to extend the proposed method to a heteroscedastic structural model.
