# Supplementary material for: DIF Statistical Inference Without Knowing Anchoring Items
Source: Psychometrika. 2023 Aug 7;88(4):1097–122. doi: 10.1007/s11336-023-09930-9 (PMC10656337; doi:10.1007/s11336-023-09930-9)
Supplement: Supplementary file 1 — (pdf 173 KB) [file 11336_2023_9930_MOESM1_ESM.pdf]

Supplementary Materials for "DIF Statistical Inference without Knowing Anchoring Items"

This file contains additional proofs of all the proposition and theorems in Section 1 and discusses asymptotic distribution of $\tilde{\Xi}$ and the implementation details of Algorithms 1 and 2 in Section 2.

1 Proofs of Propositions and Theorems

Proof of Proposition 1. Note h is differentiable for all $c \neq 0$ with,

$$\nabla h(c) = \sum_{j=1}^{J} |a_j| \cdot sign(a_j^* c - \gamma_j^*), \qquad c \neq 0.$$

Further note that $sign(a_j^* c - \gamma_j^*) = 0$ when $c = \gamma_j^*/a_j^*$ and

$$sign(a_j^* c - \gamma_j^*) > 0 \quad \text{whenever} \quad a_j^* c > \gamma_j^*, \tag{1}$$

$$sign(a_j^* c - \gamma_j^*) < 0 \quad \text{whenever} \quad a_j^* c < \gamma_j^*. \tag{2}$$

Consider the right derivative (positive directional derivative) of h at 0 from +1 direction,

$$\partial h^+(0) := \lim_{c \downarrow 0} \frac{h(c) - h(0)}{c}.$$

By the definition of right derivative of h at 0, (1) and (2), we can rewrite $\partial h^+(0)$ equivalently as follows,

$$\partial h^+(0) = \sum_{j=1}^{J} |a_j^*| \left(-I\big(\frac{\gamma_j^*}{a_j^*} > 0\big) + I\big(\frac{\gamma_j^*}{a_j^*} \leq 0\big) \right). \tag{3}$$

Similarly, define the left derivative (negative directional derivative) of h at 0 from -1 direction,

$$\partial h^-(0) := \lim_{c \uparrow 0} \frac{h(c) - h(0)}{c}.$$

By the definition of left derivative $\partial h^-(0)$, (1) and (2), we can rewrite $\partial h^-(0)$ equivalently as follows,

$$\partial h^-(0) = \sum_{j=1}^{J} |a_j^*| \left(-I\big(\frac{\gamma_j^*}{a_j^*} \geq 0\big) + I\big(\frac{\gamma_j^*}{a_j^*} < 0\big) \right). \tag{4}$$

Since h is convex, we must have $\arg\min_c h(c) = 0$ if and only if $\partial h^+(0) > 0$ and $\partial h^-(0) < 0$ (Boyd and Vandenberghe, 2004; Shor, 2012). From (3), (4) and the fact that ML1 Condition (4) is equivalent to $\arg\min_c h(c) = 0$, the result of the proposition follows directly. □

Proof of Corollary 1. By the definition of ρ^*, Condition (8) is equivalent to

$$\min_j\{|a_j^*|\} \sum_{j=1}^{J} I(\gamma_j^*/a_j^* \leq 0) > \max_j\{|a_j^*|\} \sum_{j=1}^{J} I(\gamma_j^*/a_j^* > 0).$$

For the left-hand side and right-hand side of the above inequality, we have

$$\begin{aligned} \min_j\{|a_j^*|\} \sum_{j=1}^{J} I(\gamma_j^*/a_j^* \leq 0) &< \sum_{j=1}^{J} |a_j^*| I(\gamma_j^*/a_j^* \leq 0); \\ \max_j\{|a_j^*|\} \sum_{j=1}^{J} I(\gamma_j^*/a_j^* > 0) &> \sum_{j=1}^{J} |a_j^*| I(\gamma_j^*/a_j^* > 0). \end{aligned}$$

Therefore, Condition (8) implies

$$\sum_{j=1}^{J} |a_j^*| \left(I(\gamma_j^*/a_j^* \leq 0) - I(\gamma_j^*/a_j^* > 0) \right) > 0,$$

which is (7) in Proposition 1. Similarly, we have condition (9) implies

$$\sum_{j=1}^{J} |a_j^*| \left(I(\gamma_j^*/a_j^* < 0) - I(\gamma_j^*/a_j^* \geq 0) \right) > 0.$$

which is (6) in Proposition 1. Hence, if Conditions (8) and (9) are satisfied, we have Condition (4) holds by Proposition 1.

$\square$

Proof of Theorem 1. Since MIMIC model with constraint $\gamma_1^\dagger = 0$ is identifiable, by classical asymptotic theory for MLE (van der Vaart, 2000), we have $\tilde{\Xi}$ converges in probability to $\Xi^\dagger$. That is, as $N \to \infty$, for any $\epsilon > 0$, we must have with probability tending to 1 that $|\tilde{\beta} - \beta^\dagger| \leq \epsilon$, $|\tilde{\sigma}^2 - (\sigma^2)^\dagger| \leq \epsilon$, $|\tilde{\gamma}_j - \gamma_j^\dagger| \leq \epsilon, |\tilde{a}_j - a_j^\dagger| \leq \epsilon$ and $|\tilde{d}_j - d_j^\dagger| \leq \epsilon$, for any $j = 1, ..., J$. Denote $f(c) = \sum_{j=1}^{J} |\gamma_j^\dagger - c a_j^\dagger|$ as a function of c. Similarly, denote $f_N(c) = \sum_{j=1}^{J} |\tilde{\gamma}_j - c\tilde{a}_j|$. Let $c^\dagger = \arg\min_c f(c)$ and $\hat{c} = \arg\min_c f_N(c)$, respectively. We seek to establish that $\hat{c}$ will converge in probability to $c^\dagger$ as $N \to \infty$. First note that by regularity conditions, there exists $C_1 < \infty$ such that $J, |\gamma_j^\dagger|, |a_j^\dagger| \leq C_1$. Then, there must exist $C_2 < \infty$ such that $|c^\dagger| \leq C_2$. Furthermore, note f_N is clearly continuous and convex in c, so consistency will follow if f_N can be shown to converge point-wise to f that is uniquely minimized at the true value $c^\dagger$ (typically uniform convergence is needed, but point-wise convergence of convex functions implies their uniform convergence on compact subsets). Following the model identifiability and the ML1 condition (4), $c^\dagger$ is unique. To see this, suppose for contradiction that there exist c_1 and c_2 such that $c_1 \neq c_2$ and $c_1 = \arg\min_c f(c)$ and $c_2 = \arg\min_c f(c)$. First note that $a_j^\dagger = a_j^*$ for all $j = 1, ..., J$. Then by model identifiability, there exists c_3 such that $\gamma_j^\dagger = \gamma_j^* + c_3 a_j^*$. So we have

$$c_1 = \arg\min_c \sum_{j=1}^{J} |\gamma_j^* + (c_3 - c) a_j^*|$$

and

$$c_2 = \arg\min_c \sum_{j=1}^{J} |\gamma_j^* + (c_3 - c) a_j^*|.$$

Hence, $\gamma^* = \gamma^\dagger + (c_3 - c_1) a_j^*$ and $\gamma^* = \gamma^\dagger + (c_3 - c_2) a_j^*$. If ML1 condition (4) holds, then $c_3 = c_1$

and $c_3 = c_2$. This contradicts the assumption $c_1 \neq c_2$. Hence, $c^\dagger$ must be unique. For any $|c| \leq C_2$,

$$
\begin{aligned}
&|f_N(c) - f(c)| \\
&= \Big| \sum_{j=1}^{J} \Big(|\tilde{\gamma}_j - c\tilde{a}_j| - |\gamma_j^\dagger - c a_j^\dagger| \Big) \Big| \\
&\leq \Big| \sum_{j=1}^{J} \Big(|(\tilde{\gamma}_j - c\tilde{a}_j) - (\gamma_j^\dagger - c a_j^\dagger)| \Big) \Big| \\
&= \Big| \sum_{j=1}^{J} \Big(|(\tilde{\gamma}_j - \gamma_j^\dagger) + c(a_j^\dagger - \tilde{a}_j)| \Big) \Big| \\
&\leq \sum_{j=1}^{J} \Big(|\tilde{\gamma}_j - \gamma_j^\dagger| + |c| \cdot |a_j^\dagger - \tilde{a}_j| \Big) \\
&\leq J\epsilon + |c|\epsilon. \\
&\leq (C_1 + C_2)\epsilon.
\end{aligned}
$$

Take $\epsilon_1 = (C_1 + C_2)\epsilon$, it follows that for any fixed $|c| \leq C_2$, $P\big(|f_N(c) - f(c)| \leq \epsilon_1\big) \to 1$ as $N \to \infty$. Moreover, following from the uniqueness of $c^\dagger$ and the continuity and the convexity of $f_N(\cdot)$ in c, we must have $|\hat{c} - c^\dagger| = o_P(1)$ as $N \to \infty$.

Note that $\hat{\beta} = \tilde{\beta} + \hat{c}$, $\hat{\sigma}^2 = \tilde{\sigma}^2$, $\hat{\gamma}_j = \tilde{\gamma}_j - \hat{c}\tilde{a}_j$, $\hat{a}_j = \tilde{a}_j$, $\hat{d}_j = \tilde{d}_j$ for all $j = 1, ..., J$. From the model identifiability and the ML1 condition (4), we know that $\beta^* = \beta^\dagger + c^\dagger$, $(\sigma^2)^* = (\sigma^2)^\dagger$, $\gamma_j^* = \gamma_j^\dagger - c^\dagger a_j^\dagger$, $a_j^* = a_j^\dagger$, $d_j^* = d_j^\dagger$ for all $j = 1, ..., J$. Since $|\hat{c} - c^\dagger| = o_P(1)$, $|\tilde{\beta} - \beta^\dagger| = o_P(1)$, $|\tilde{\sigma}^2 - (\sigma^2)^\dagger| = o_P(1)$, $|\tilde{\gamma}_j - \gamma_j^\dagger| = o_P(1), |\tilde{a}_j - a_j^\dagger| = o_P(1), |\tilde{d}_j - d_j^\dagger| = o_P(1)$ as $N \to \infty$, it follows directly from the Slutsky's Theorem that $|\hat{\beta} - \beta^*| = o_P(1), |\hat{\sigma}^2 - (\sigma^2)^*| = o_P(1), |\hat{\gamma}_j - \gamma_j^*| = o_P(1), |\hat{a}_j - a_j^*| = o_P(1)$, $|\hat{d}_j - d_j^*| = o_P(1)$ as $N \to \infty$. □

2 Asymptotic Distribution of $\tilde{\Xi}$

Since the model is identifiable with constraint $\gamma_1^\dagger = 0$ and all the regularity conditions in Theorem 5.39 of van der Vaart (2000) are satisfied, hence, by Theorem 5.39 in van der Vaart (2000),

$\tilde{\Xi} \to \mathrm{N}(\Xi^\dagger, \Sigma^*)$ in distribution as $N \to \infty$. In practice, we use the inverse of the observed Fisher information matrix, denoted by $\hat{\Sigma}_N$, which is a consistent estimator of Σ^*, to draw Monte Carlo samples. Below, we give procedures to evaluate $\hat{\Sigma}_N$ from the marginal log-likelihood.

Following the notations in the main article, we first provide the complete data log-likelihood function,

$$l(\Xi; Y) = \sum_{i=1}^{N} \Big[\log \Big\{ \frac{1}{\sqrt{2\pi(1_{\{x_i=0\}} + \sigma^2 1_{\{x_i=1\}})}} \exp\Big(\frac{-(\theta_i - \beta x_i)^2}{2(1_{\{x_i=0\}} + \sigma^2 1_{\{x_i=1\}})} \Big) \Big\} + \sum_{j=1}^{J} \big\{ y_{ij}(a_j\theta_i + d_j + \gamma_j x_i) - \log(1 + \exp\{a_j\theta_i + d_j + \gamma_j x_i\}) \big\} \Big].$$

Since θ_i is considered as a random variable such that $\theta_i \mid x_i \sim \mathrm{N}(\beta x_i, 1_{\{x_i=0\}} + \sigma^2 1_{\{x_i=1\}})$, so we will work with the marginal log-likelihood function,

$$mll(\Xi; Y) = \sum_{i=1}^{N} \log \Big\{ \int \Big(\prod_{j=1}^{J} \frac{\exp(y_{ij}(a_j\theta_i + d_j + \gamma_j x_i))}{1 + \exp(a_j\theta_i + d_j + \gamma_j x_i)} \Big) \frac{1}{\sqrt{2\pi}} \exp\Big(\frac{-(\theta_i - \beta x_i)^2}{2(1_{\{x_i=0\}} + \sigma^2 1_{\{x_i=1\}})} \Big) d\theta_i \Big\}.$$

Note that the observed Fisher information matrix $I(\Xi)$ cannot be directly obtained from the $mll(\Xi; Y)$ due to the intractable integral. Instead, we apply the Louis Identity (Louis, 1982) to evaluate the observed Fisher information matrix. Let $S(\Xi; Y)$ and $B(\Xi; Y)$ denote the gradient vector and the negative of the hessian matrix of the complete data log-likelihood function, respectively. Then by the Louis Identity, $I(\Xi)$ can be expressed as

$$I(\Xi) = \mathbf{E}_\theta[B(\Xi; Y) \mid Y] - \mathbf{E}_\theta[S(\Xi; Y) S(\Xi; Y)^T \mid Y] + \mathbf{E}_\theta[S(\Xi; Y) \mid Y] \mathbf{E}_\theta[S(\Xi; Y) \mid Y]^T.$$

Denote $p_{ij} = \exp\{y_{ij}(a_j\theta_i + d_j + \gamma_j x_i)\}/[1 + \exp\{y_{ij}(a_j\theta_i + d_j + \gamma_j x_i)\}]$. Then, in particular,

$$\begin{aligned}
S(\Xi;Y) =& \frac{\partial l(\Xi;Y)}{\partial \Xi} \\
=& \Big\{\frac{\partial l(\Xi;Y)}{\partial \beta}, \frac{\partial l(\Xi;Y)}{\partial \sigma^2}, ..., \frac{\partial l(\Xi;Y)}{\partial a_j}, ..., \frac{\partial l(\Xi;Y)}{\partial d_j}, ..., \frac{\partial l(\Xi;Y)}{\partial \gamma_j}, ...\Big\} \\
=& \Big\{\frac{\sum_{i=1}^N x_i(\theta_i - \beta)}{\sigma^2}, \frac{\sum_{i=1}^N x_i(\theta_i - \beta)^2}{2\sigma^4} - \frac{\sum_{i=1}^N x_i}{2\sigma^2}, ..., \\
& \sum_{i=1}^N \theta_i(y_{ij} - p_{ij}), ..., \sum_{i=1}^N (p_{ij} - y_{ij}), ..., \sum_{i=1}^N x_i(y_{ij} - p_{ij})\Big\}.
\end{aligned}$$

Furthermore, note that $B(\Xi;Y) = -\partial^2 l(\Xi;Y)/\partial\Xi\partial\Xi^T$ is a $(3J+2)$ by $(3J+2)$ matrix with the only non-zero entries,

$$\begin{aligned}
\frac{\partial^2 l(\Xi;Y)}{\partial \beta^2} &= -\frac{\sum_{i=1}^N x_i}{\sigma^2}, \\
\frac{\partial^2 l(\Xi;Y)}{\partial (\sigma^2)^2} &= -\frac{\sum_{i=1}^N x_i(\theta_i - \beta)^2}{\sigma^6} + \frac{\sum_{i=1}^N x_i}{2\sigma^4}, \\
\frac{\partial^2 l(\Xi;Y)}{\partial \beta \partial \sigma^2} &= -\frac{\sum_{i=1}^N x_i(\theta_i - \beta)}{\sigma^4}, \\
\frac{\partial^2 l(\Xi;Y)}{\partial a_j^2} &= -\sum_{i=1}^N \theta_i^2 p_{ij}(1 - p_{ij}), \\
\frac{\partial^2 l(\Xi;Y)}{\partial d_j^2} &= -\sum_{i=1}^N p_{ij}(1 - p_{ij}), \\
\frac{\partial^2 l(\Xi;Y)}{\partial \gamma_j^2} &= -\sum_{i=1}^N x_i^2 p_{ij}(1 - p_{ij}), \\
\frac{\partial^2 l(\Xi;Y)}{\partial a_j \partial d_j} &= \sum_{i=1}^N \theta_i p_{ij}(1 - p_{ij}), \\
\frac{\partial^2 l(\Xi;Y)}{\partial a_j \partial \gamma_j} &= -\sum_{i=1}^N \theta_i x_i p_{ij}(1 - p_{ij}), \\
\frac{\partial^2 l(\Xi;Y)}{\partial d_j \partial \gamma_j} &= \sum_{i=1}^N x_i p_{ij}(1 - p_{ij}).
\end{aligned}$$

In practice, we can use Gaussian quadrature method to approximate the expectation of these terms so as to obtain $\hat{I}(\tilde{\Xi})$. Then $\hat{\Sigma}_N$ can be evaluated with $\hat{\Sigma}_N = \hat{I}^{-1}(\tilde{\Xi})$. This then enables Step 1 of Algorithm 1, where Monte Carlo samples of $\Xi^\dagger$ can be simulated from $\mathrm{N}(\tilde{\Xi}, \hat{\Sigma}_N)$.

References

Boyd, S. and Vandenberghe, L. (2004). *Convex optimization.* Cambridge University Press, Cambridge, UK.

Louis, T. A. (1982). Finding the observed information matrix when using the EM algorithm. *Journal of the Royal Statistical Society: Series B (Methodological)*, 44(2):226–233.

Shor, N. Z. (2012). *Minimization methods for non-differentiable functions.* Springer, New York, NY.

van der Vaart, A. W. (2000). *Asymptotic statistics.* Cambridge University Press, Cambridge, UK.
